# Overelaborated synaptic architecture and reduced synaptomatrix glycosylation in a *Drosophila* classic galactosemia disease model

**DOI:** 10.1242/dmm.017137

**Published:** 2014-10-17

**Authors:** Patricia Jumbo-Lucioni, William Parkinson, Kendal Broadie

**Affiliations:** Department of Biological Sciences, Kennedy Center for Research on Human Development, Vanderbilt University, Nashville, TN 37232, USA.

**Keywords:** Congenital disorder of glycosylation (CDG), *sugarless*, Galactokinase, Synaptogenesis, Trans-synaptic signaling, WNT, HSPG, Neuromuscular junction

## Abstract

Classic galactosemia (CG) is an autosomal recessive disorder resulting from loss of galactose-1-phosphate uridyltransferase (GALT), which catalyzes conversion of galactose-1-phosphate and uridine diphosphate (UDP)-glucose to glucose-1-phosphate and UDP-galactose, immediately upstream of UDP–N-acetylgalactosamine and UDP–N-acetylglucosamine synthesis. These four UDP-sugars are essential donors for driving the synthesis of glycoproteins and glycolipids, which heavily decorate cell surfaces and extracellular spaces. In addition to acute, potentially lethal neonatal symptoms, maturing individuals with CG develop striking neurodevelopmental, motor and cognitive impairments. Previous studies suggest that neurological symptoms are associated with glycosylation defects, with CG recently being described as a congenital disorder of glycosylation (CDG), showing defects in both N- and O-linked glycans. Here, we characterize behavioral traits, synaptic development and glycosylated synaptomatrix formation in a *GALT*-deficient *Drosophila* disease model. Loss of *Drosophila GALT* (*dGALT*) greatly impairs coordinated movement and results in structural overelaboration and architectural abnormalities at the neuromuscular junction (NMJ). Dietary galactose and mutation of galactokinase (*dGALK*) or UDP-glucose dehydrogenase (*sugarless*) genes are identified, respectively, as critical environmental and genetic modifiers of behavioral and cellular defects. Assaying the NMJ extracellular synaptomatrix with a broad panel of lectin probes reveals profound alterations in *dGALT* mutants, including depletion of galactosyl, N-acetylgalactosamine and fucosylated horseradish peroxidase (HRP) moieties, which are differentially corrected by *dGALK* co-removal and *sugarless* overexpression. Synaptogenesis relies on trans-synaptic signals modulated by this synaptomatrix carbohydrate environment, and *dGALT*-null NMJs display striking changes in heparan sulfate proteoglycan (HSPG) co-receptor and Wnt ligand levels, which are also corrected by *dGALK* co-removal and *sugarless* overexpression. These results reveal synaptomatrix glycosylation losses, altered trans-synaptic signaling pathway components, defective synaptogenesis and impaired coordinated movement in a CG neurological disease model.

## INTRODUCTION

Classic galactosemia (CG; OMIM 230400) results from loss of galactose-1-phosphate uridyltransferase (GALT), the second enzyme in the Leloir pathway, which acts immediately downstream of galactokinase (GALK), the initial enzyme (McCorvie and Timson, 2011). GALT maintains the balance between uridine diphosphate (UDP)-glucose (glc), -galactose (gal), –N-acetylgalactosamine (GalNac) and –N-acetylglucosamine (GlcNac) (Frey, 1996). Levels of these four UDP-sugars are rate-limiting for the biosynthesis of glycoproteins and proteoglycans (Freeze and Elbein, 2009), which form the foundation of the extracellular synaptomatrix of the synaptic cleft and perisynaptic space (Dani and Broadie, 2012). UDP-glc dehydrogenase is encoded by *Drosophila sugarless* (*sgl*) (Häcker et al., 1997; Toyoda et al., 2000), and *sgl* mutations compromise biosynthesis of the heparan sulfate proteoglycan (HSPG) co-receptor Dally-like protein (Dlp), which is known to regulate trans-synaptic signaling of the Wnt protein Wingless (Wg); such signaling drives neuromuscular junction (NMJ) synaptogenesis (Dani et al., 2012). These studies implicate a core pathway involving GALT, GALK and Sgl in the regulation of HSPG co-receptor control of Wnt signaling during NMJ synapse formation, and indicate that disruption of this pathway is a potential causal mechanism underlying CG neuropathology.

Acute CG neonatal symptoms are alleviated by dietary galactose restriction (Jumbo-Lucioni et al., 2012), but maturing individuals with CG develop substantial neurodevelopmental, motor and cognitive impairments (Ridel et al., 2005). After >50 years of research, there is still no mechanistic understanding of these chronic neurological symptoms. However, a long-term and extensive body of studies documents glycosylation defects in individuals with CG (Haberland et al., 1971; Petry et al., 1991; Charlwood et al., 1998; Liu et al., 2012). Galactose is a major component of complex carbohydrates in glycoproteins and glycolipids in the nervous system, and defective glycosylation impairs neurodevelopment and neurological function (Freeze et al., 2012). In particular, the heavily glycosylated NMJ synaptomatrix plays crucial roles in synaptogenesis during normal development, and its disruption is implicated in numerous heritable disease states (Dani and Broadie, 2012). For example, glycosylation defects are causal in numerous muscular dystrophies (MDs) and congenital disorders of glycosylation (CDGs) that are characterized by severe neurological impairments (Muntoni et al., 2008; Freeze, 2013).

We recently conducted a *Drosophila* screen of glycogenes via RNAi knockdown of N/O-linked glycans, glycosaminoglycans, glycosyltransferases and glycan-binding lectins to test the effects on NMJ structure and function (Dani et al., 2012). This screen identified *Drosophila GALT* (*dGALT*) as a potent regulator of NMJ architecture. We therefore set forth to characterize synaptic morphology and glycosylated synaptomatrix composition in the recently established *Drosophila* CG disease model (*dGALT* deficiency) (Kushner et al., 2010), and to identify glycan mechanisms driving synaptogenic defects. We found that *dGALT* nulls exhibit a profoundly altered carbohydrate landscape within the NMJ synaptomatrix, accompanied by loss of the HSPG co-receptor Dlp and extracellular accumulation of Wg ligand. Crucially, synaptomatrix defects were differentially corrected by *dGALK* co-removal and *sgl* overexpression. Consistently, we found that *dGALK* removal and *sgl* overexpression in *dGALT* mutants corrected both the motor defects and NMJ architectural abnormalities resulting from *dGALT* loss of function. We conclude that *dGALT*, *dGALK* and *sgl* define a genetic pathway regulating synaptomatrix glycosylation state to modulate components of a Wnt trans-synaptic signaling pathway, and thereby control NMJ synaptic morphogenesis to support coordinated movement.

**TRANSLATIONAL IMPACT**

**Clinical issue**

Classic galactosemia (CG) results from complete or almost complete loss of galactose-1-phosphate uridyltransferase (GALT), the second enzyme in the Leloir pathway of galactose metabolism. GALT catalyzes the generation of a glucose precursor and maintains the balance between uridine diphosphate (UDP) sugars, the obligate sugar donors for the synthesis of glycoproteins and glycolipids (glycosylation), which are important components of cell membranes and extracellular matrix. Dietary galactose restriction alleviates neonatal lethal symptoms of CG, but affected individuals still develop substantial neurological complications (including motor and cognitive impairments) of unknown etiology. CG has recently been classified as a congenital disorder of glycosylation (CDG), and glycosylation defects have been suggested as the underlying mechanism for the chronic neurological disease symptoms. Beyond the single CG condition, more than 60 other CDGs have been identified so far. Neurological impairments are common in these CDG disease states, but effective treatments are not yet available.

**Results**

A recent *Drosophila* screen of glycosylation-related genes identified GALT as a potent regulator of the structure of the neuromuscular junction (NMJ; the synapse connecting motor neurons and muscle fibers). To extend this discovery, GALT-deficient *Drosophila* (*dGALT* nulls; a CG model) have been used to characterize movement behavior, NMJ synapse structure and function, and the carbohydrate composition of the synapse extracellular environment (i.e. synaptomatrix) in the disease. The results show that loss of GALT activity impairs coordinated movement, causes structural synapse overelaboration, profoundly changes the carbohydrate composition of the NMJ synaptomatrix, and alters the Wnt trans-synaptic co-receptor and ligand abundance (known to control synaptic morphogenesis at the NMJ). In double mutants, the combination of GALT deficiency either with loss of galactokinase (GALK; an enzyme that acts upstream of GALT) or overexpression of UDP-glucose dehydrogenase (an enzyme that acts downstream of GALT; encoded by *sugarless*) corrects the glycosylation defects, the NMJ architecture alterations and the movement impairments associated with *GALT* loss in this *Drosophila* CG model.

**Implications and future directions**

NMJ defects likely underlie the pathogenesis of movement disorders frequently reported in CG-affected individuals, and might also account for similar impairments that characterize other CDG disease states. The findings reported here are the first to reveal NMJ synaptic glycan loss, synaptic architecture defects and trans-synaptic Wnt co-receptor and ligand defects as potential causal factors for the well-known CG-related movement disabilities. Furthermore, the results presented here suggest that targeting galactokinase and UDP-glucose dehydrogenase could modify CG outcome and represent a potential therapeutic approach in this disease. Future investigations are needed to advance the translation of findings on CG-related candidate genes into the development of novel drugs, and to extend this approach to the study of other CDG diseases.

## RESULTS

### Impaired coordinated movement of *dGALT* nulls is rescued by human *GALT* expression

Evidence amassed over decades from individuals with CG documents common movement defects (Waggoner et al., 1990; Schweitzer et al., 1993; Kaufman et al., 1995; Hughes et al., 2009; Rubio-Agusti et al., 2013). Similarly, *dGALT*-null mutants display defects in startle-induced, geo-negative climbing behavior (Ryan et al., 2012). To assay locomotion defects directly, we first assayed daily motor activity levels in individual animals using the *Drosophila* Activity Monitoring (DAM) system, which measures movement disruption of an infrared beam (Chiu et al., 2010). Adult animals aged 3–5 days were compared between *dGALT^ΔAP2^* (null) and *dGALT^C2^* (precise-excision genetic control) flies. Both genotypes were entrained to 12:12-hour light:dark cycles for 2 days, and then activity counts were recorded for ≥3 days. *dGALT*-null mutants were significantly (*P*<0.01) movement-impaired compared with controls (20.2±1.8 versus 27.7±2.1 counts/hour during the light period; *n*≥20 for each genotype). These results show that removal of *dGALT* strongly reduces locomotor activity in this CG model.

**Fig. 1.**
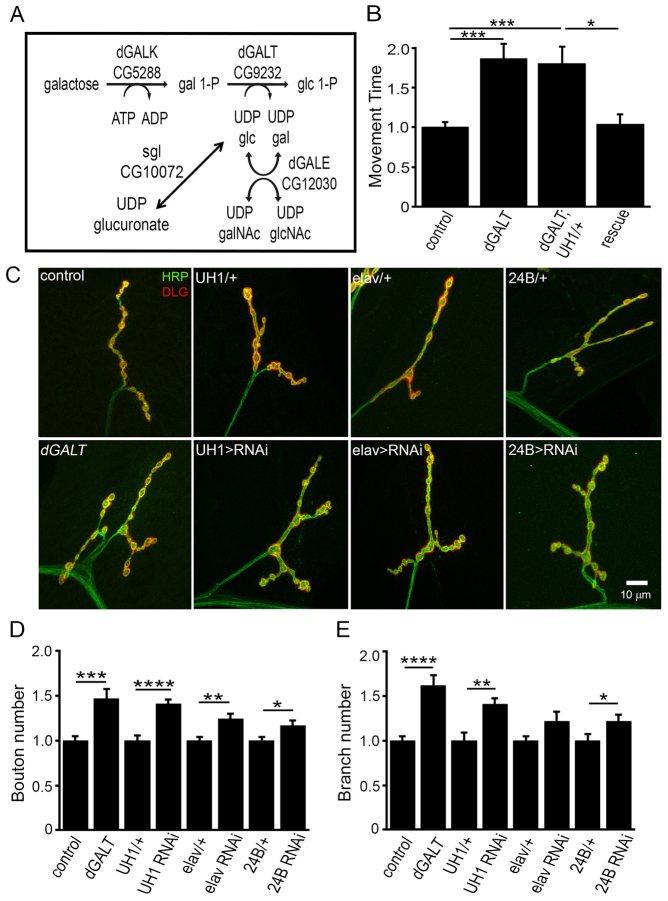
**Loss of *dGALT* impairs coordinated movement and disrupts NMJ architecture.** (A) Schematic diagram of the Leloir pathway. Galactose is phosphorylated by *Drosophila* galactokinase (dGALK) and then *Drosophila* galactose-1-P uridylyltransferase (dGALT) catalyzes the synthesis of glucose-1-P (glc-1-P) and UDP-galactose (UDP-gal) from UDP-glucose (UDP-glc) and galactose-1P (gal-1-P). UDP-glucose is a substrate for UDP-glucose dehydrogenase [encoded by *sugarless* (*sgl*)], catalyzing conversion to UDP-glucuronate, essential for proteoglycan biosynthesis. dGALE, uridine diphosphate galactose-4-epimerase. (B) Fold differences in the time required (controls set at 1) for wandering L3 to rollover from inverted to upright position for control (*dGALT^C2^*), *dGALT* null (*dGALT^ΔAP2^*), transgenic rescue (*dGALT^ΔAP2^; UH1-Gal4/UAS-hGALT*) and rescue control (*dGALT^ΔAP2^; UH1-Gal4/+*). Data are normalized to respective control. (C) Representative NMJs imaged with anti-horseradish-peroxidase (HRP; green) and anti-Discs-large (DLG; red) in wandering L3 in *dGALT* mutants (bottom row) with complete loss (*dGALT^ΔAP2^*), ubiquitous (*UH1-Gal4* driven) and tissue-specific (i.e. *elav*- or *24-Gal4*-driven) knockdown. Respective controls for each condition are shown in the top row. (D,E) Quantification of differences in NMJ bouton (D) and branch (E) number for all genotypes, normalized to appropriate controls. Sample size: ≥ten animals per genotype. Error bars show s.e.m. with significance indicated: *P<0.05, **P<0.01, ***P<0.001, ****P<0.0001.

We next turned to the well-characterized movement behaviors in *Drosophila* larvae (Heckscher et al., 2012). Bilateral coordinated movement can be quantified with a rollover assay (Bodily et al., 2001; Pan et al., 2008). We tested wandering L3 larvae that had complete loss of *dGALT* (*dGALT^ΔAP2^*, *n*=37 and *dGALT^ΔAP2^*/Df, *n*=34), and compared with a genetic control (*dGALT^C2^*, *n*=53). Compared with the righting time of controls (17.20±1.03 s), both *dGALT* (30.95±2.59 s; *P*<0.001) and *dGALT*/Df (27.81±3.04 s; *P*<0.05) animals were significantly slower and obviously less coordinated (Fig. 1B). [Note that, in all graphs shown in the figures, fold change compared with control values (set at 1) are shown.] There was no significant difference between the two null conditions. To confirm that the movement defect is mediated by *dGALT* loss, we next drove wild-type human *GALT* (UAS-*hGALT*) in the *dGALT^ΔAP2^*-null background (Fig. 1B; ‘rescue’, *n*=14). Transgenic *hGALT* expression in the *dGALT^ΔAP2^*-null background completely rescued normal coordinated movement compared with the driver-alone control (*dGALT*; UH1-*Gal4*/+, *n*=15; *P*=0.006), which was indistinguishable from homozygous *dGALT^ΔAP2^* mutants (*dGALT*; Fig. 1B). These results demonstrate that dGALT activity is necessary for proper coordinated movement, and that this requirement is functionally conserved with human GALT. Because irregularities in NMJ architecture can cause locomotor impairments, we next characterized synaptic structure under conditions of intact, complete loss of and partial loss of dGALT activity.

### Loss of *dGALT* causes striking structural defects at the NMJ synapse

Our recent *Drosophila* RNAi screen identified that *dGALT* is required in NMJ morphological synaptogenesis (Dani et al., 2012). The glutamatergic NMJ, which drives coordinated movement, has been well characterized in *Drosophila* larvae (Andlauer and Sigrist, 2012; Collins and DiAntonio, 2007), and the structure and function of this synaptic terminal is highly dependent on appropriate expression of extracellular glycans (Dani and Broadie, 2012). We therefore hypothesized that defects in NMJ development underlie motor impairments in *dGALT* mutants (Xia et al., 2012; Courchesne et al., 2011). Axonal growth properties, branch formation and synaptic bouton differentiation together shape the complex three-dimensional synaptic architecture (Broadie et al., 2011; Nahm et al., 2013). NMJs from wandering L3 larvae were labeled with anti-horseradish-peroxidase (HRP; presynaptic) and anti-DLG (postsynaptic) in nine genotypes: genetic background control (*dGALT^C2^*; precise excision), homozygous-null *dGALT^ΔAP2^* and *dGALT^ΔAP2^*/Df, and ubiquitous *UH1*-, neuronal *elav*- and muscle *24B-Gal4*-driven *dGALT*-RNAi with their respective controls (*UH1-Gal4*/+, *elav-Gal4*/+ and *24B-Gal4*/+, respectively). Confocal images for representative genotypes are shown in Fig. 1C.

*dGALT* nulls as well as ubiquitous (*UH1-Gal4*) and tissue-targeted (*elav-Gal4* and *24B-Gal4*) *dGALT*-knockdown NMJs all exhibited obvious synaptic structural overelaboration (Fig. 1C, bottom row), compared with matched genetic controls (Fig. 1C, top row). Synaptic bouton number was significantly increased in all four mutant conditions (Fig. 1D). The increase in bouton number was highly significant in both *dGALT*-null conditions (*dGALT*: 39.4±2.7, *n*=14, *P*<0.001; *dGALT*/Df: 42.5±2.3, *n*=17, *P*<0.001) compared with controls (28.1±1.5, *n*=32), as well as in the ubiquitous *dGALT* RNAi-knockdown condition (*UH1*-driven: 36.7±1.4, *n*=20, *P*<0.0001) compared with controls (26.6 ±1.9, *n*=13; Fig. 1D). Both neuronal (*elav-Gal4*) and muscle (*24B-Gal4*)-driven *dGALT* RNAi showed more modest, but significant, increases in bouton number (34.8±1.7, *n*=28, *P*=0.002 and 31.0±1.6, *n*=20, *P*=0.03, respectively) compared with driver-alone controls (28.0±1.2, *n*=31 and 26.5±1.2, *n*=17, respectively; Fig. 1D). In addition, loss of dGALT activity increased synapse branch number in *dGALT* nulls (*P*<0.0001) and with ubiquitous *dGALT* RNAi knockdown (*UH1-Gal4*-driven; *P*=0.004; Fig. 1E). Finally, although branch number was increased with neuronal *dGALT* knockdown compared with that in controls, this trend was only significant with *dGALT* knockdown in muscle (Fig. 1E). Importantly, transgenic expression of human *GALT* in the homozygous *dGALT^ΔAP2^* null (*n*=9) fully rescued both bouton and branch numbers. These results demonstrate that *dGALT* is necessary for sculpting NMJ synaptic architecture, and that this requirement is fully met with transgenic expression of human *GALT*.

To test NMJ neurotransmission strength, the motor nerve was stimulated with a glass suction electrode while recording from the voltage-clamped muscle (Parkinson et al., 2013). Excitatory junction current (EJC) recordings were made at suprathreshold stimulation levels at a 0.5 Hz frequency. Four genotypes were compared: genetic background control (*dGALT^C2^*; precise excision) to homozygous-null mutant (*dGALT^ΔAP2^*; imprecise excision), and *UH1-Gal4*-driven UAS-*dGALT*-RNAi to *UH1-Gal4*/+-alone transgenic control. More than ten NMJs from ≥five different animals were recorded for each of the four genotypes. Mean EJC amplitudes were not significantly different in *dGALT* nulls compared with controls (419.2±23.9 versus 403.2±17.5 nA; *P*>0.05; data not shown). Similarly, ubiquitous *dGALT* knockdown animals also displayed similar synaptic strength to controls (369.9±19.5 versus 328.6±27.5 nA; *P*>0.05; data not shown). Although there was a trend towards increased neurotransmission in both comparisons, neither change was significant. We therefore conclude that *dGALT* does not significantly alter neurotransmission strength at the NMJ, but rather plays a specific role in controlling synaptic architecture, which we therefore focused on in subsequent studies. Because exposure to dietary galactose is a well-recognized modifier of acute outcome in CG (Jumbo-Lucioni et al., 2013), we next characterized locomotion and NMJ structural defects with galactose dietary supplementation.

### High-galactose diet phenocopies *dGALT*-mutant defects

**Fig. 2.**
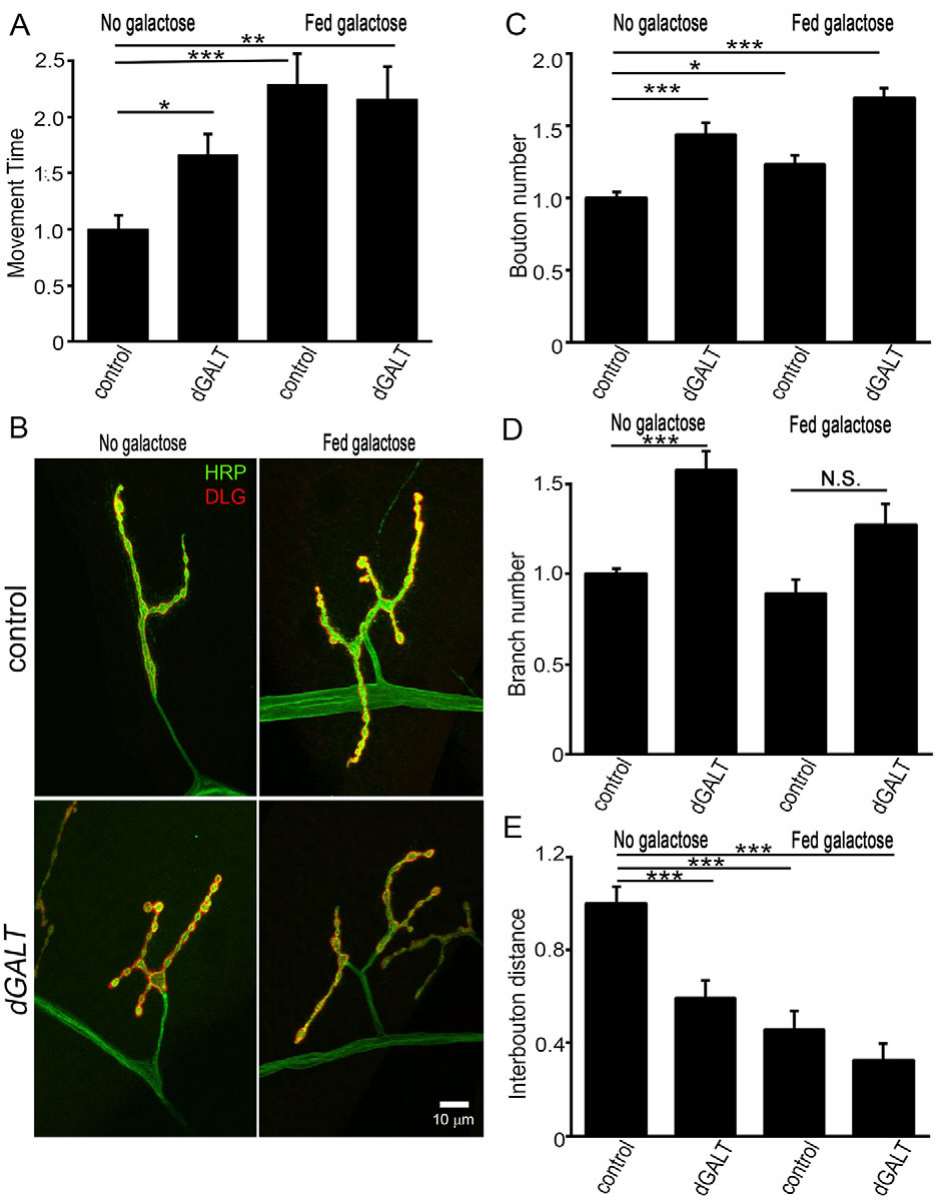
**High-galactose diet phenocopies *dGALT*-null movement and NMJ defects.** (A) Differences in the time required for wandering L3 larvae to rollover from inverted to upright position: control (*dGALT^C2^*) and *dGALT*-null (*dGALT^ΔAP2^*) flies were fed food that was either galactose-free or supplemented with 200 mM galactose. (B) Representative NMJs imaged with anti-horseradish peroxidase (HRP; green) and anti-Discs-large (DLG; red) in wandering L3 for the above four conditions. (C–E) Quantification of synaptic bouton number (C), branch number (D) and inter-bouton spacing distance (E) for all four conditions, normalized to appropriate controls. Sample size: ≥ten animals per genotype. Error bars show s.e.m. with significance indicated: *P<0.05, **P<0.01, ***P<0.001.

Dietary galactose is a well-recognized modifier of CG patient acute outcome (Jumbo-Lucioni et al., 2012). However, there is controversy regarding galactose restriction in preventing chronic complications (Hughes et al., 2009; Jumbo-Lucioni et al., 2012), particularly in preventing CG neurological symptoms, and whether supplementation or restriction are important environmental modifiers of disease outcome. We therefore next assayed whether galactose feeding in the *Drosophila* CG model modifies movement and NMJ phenotypes. Genetic background control (*dGALT^C2^*) and homozygous-null (*dGALT^ΔAP2^*) animals were raised on either (1) normal molasses-based food containing negligible galactose levels (Kushner et al., 2010; Jew et al., 1990), or (2) 200 mM galactose-supplemented food. Wandering L3 larvae were collected to first characterize movement (Fig. 2A). We found that galactose feeding severely compromised the ability of controls to display coordinated motor activity. In rollover assays, controls raised in galactose-enriched food took as much time as *dGALT* nulls raised in the same conditions to complete the movement (33.0±4 s, *n*=27 versus 31.1±4.2 s, *n*=21, respectively), and ~120% more time than control animals fed galactose-free food (*n*=24, *P*<0.001; Fig. 2A). Moreover, *dGALT*-null movement defects were not worsened by a high-galactose diet, and were not significantly different with or without dietary galactose (24.7±2.7 s, *n*=21 versus 31.1±4.2 s, *n*=26; Fig. 2A). Thus, galactose feeding in wild-type animals phenocopies *dGALT*-null movement defects.

The same general conclusion applies to NMJ structural defects. Galactose-fed control and homozygous-*dGALT*-null larvae, and homozygous-*dGALT*-null larvae raised in the absence of galactose, displayed supernumerary boutons (28.4±1.4, *n*=13; 35.0±2.1, *n*=12; 35.1±1.5, *n*=18, respectively; Fig. 2B,C); these values were all significantly greater than those for control animals raised in galactose-free conditions (23.7±1.2 boutons, *n*=21; *P*<0.05 compared with galactose-fed controls; Fig. 2B,C). In galactose-free conditions, mutant NMJs exhibited significantly more branches (3.56±0.20, *n*=18) than did controls (2.45±0.11, *n*=21), and dietary galactose did not alter these values for either genotype (control 2.27±0.17, *n*=13; *dGALT* 3.42±0.31, *n*=12; Fig. 2D). Furthermore, although we did not find significant differences in NMJ axon cumulative length, *dGALT* nulls exhibited significantly reduced interbouton distances compared with controls (0.57±0.06 versus 0.87±0.07 μm; *P*<0.001; Fig. 2E). Elevated dietary galactose similarly reduced the distance between synaptic boutons in controls (0.29±0.03 μm; *P*<0.001) and eliminated the difference with null mutants (0.28±0.04 μm; Fig. 2E). Taken together, these results show that the movement and NMJ phenotypes of the *Drosophila* CG model are generally not worsened by high dietary galactose, but rather that high galactose intake in wild-type animals phenocopies *dGALT* movement and most NMJ structural deficits.

It has been previously shown that a high-galactose diet fed to wild-type larvae causes accumulation of galactose-1-phosphate (gal-1-P) (Kushner et al., 2010), which was previously shown to alter the UDP-sugar balance (Lai et al., 2003). To further test whether motor behavior and NMJ structural defects occurring in galactose-fed conditions are linked to gal-1-P accumulation, *dGALK* nulls (*dGALK^ΔEXC9^*), with undetectable enzymatic activity, were raised either on food supplemented with 200 mM galactose or on normal food. Galactose-fed mutants took as much time as unsupplemented mutants to rollover from an inverted to an upright position (8.84±0.90 s versus 9.5±1.57 s; *n*≥8). Similarly, both NMJ branch number and interbouton distances were not significantly different in the two conditions (data not shown). Animals displayed 2.0±0.19 (*n*=8) and 2.2±0.17 (*n*=15) branches under galactose-free and galactose-fed conditions, respectively. Interbouton distance was 0.81±0.08 μm (*n*=15) and 0.77±0.06 μm (*n*=8) with and without galactose feeding, respectively. However, galactose-fed *dGALK* mutants displayed excess NMJ boutons compared with unsupplemented animals (23.27±1.23 versus 14.57±1.02; *n*≥8, *P*<0.0001) and significantly greater NMJ cumulative length (113.08±6.04 μm versus 81.65±4.36 μm, *n*≥8, *P*=0.002). Because a high-galactose diet can alter glycosylation status, and abnormal glycosylation of the extracellular environment at the NMJ is well known to modulate synaptogenesis (Dani and Broadie, 2012), we next characterized the glycosylated synaptomatrix composition of the NMJ.

### dGALT activity shapes the glycosylated synaptomatrix composition of the NMJ

**Fig. 3.**
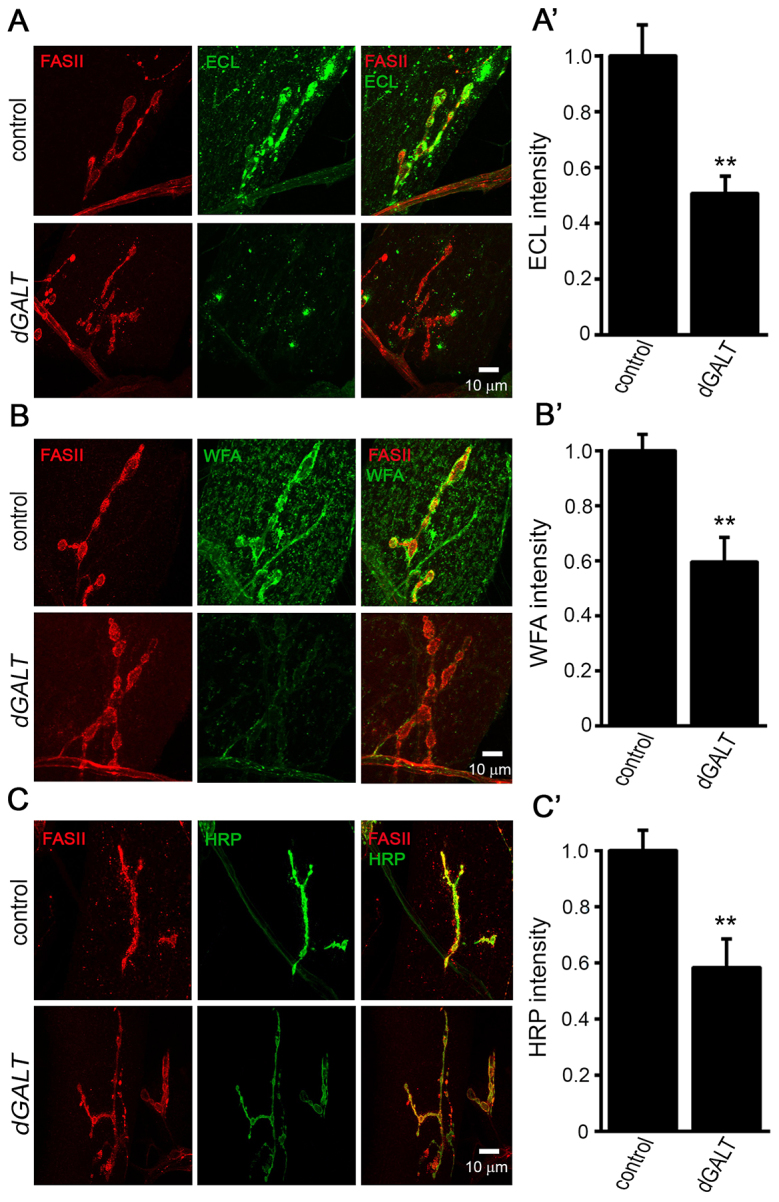
**Loss of dGALT activity compromises the NMJ glycosylated synaptomatrix.** Representative wandering L3 NMJs imaged with anti-Fasciclin-II (FASII; red) in all cases and co-labeled with lectins (A) *Erythrina cristagalli* (ECL; green), (B) *Wisteria floribunda* (WFA; green) and (C) anti-horseradish-peroxidase (HRP; green) in genetic controls (precise-excision *dGALT^C2^*) and *dGALT* nulls (imprecise-excision *dGALT^ΔAP2^*). Quantification shows normalized ECL (≥15 NMJs) (A′), WFA (≥19 NMJs) (B′) and anti-HRP (≥16 NMJs) (C′) intensities. Error bars show s.e.m. with significance indicated as **P<0.01.

Glycosylation defects in plasma and tissue samples have long been reported in individuals with CG (Haberland et al., 1971; Jaeken et al., 1992; Sturiale et al., 2005). These glycan errors persist even after prolonged dietary galactose restriction, and have therefore been suggested to account for chronic neurological symptoms in such individuals (Charlwood et al., 1998). Importantly, abnormally glycosylated synaptomatrix at the *Drosophila* NMJ is well known to alter synapse formation (Dani and Broadie, 2012), and therefore provides a probable mechanistic basis for the synaptic architecture defects manifested in *dGALT*-null mutants (Figs 1, 2). To test this hypothesis, we next assayed the NMJ synaptomatrix with a panel of lectins labeling (1) terminal galactosyl residues [Peanut agglutinin (PNA) and *Erythrina cristagalli* lectin (ECL)], (2) N-acetylgalactosamine residues [*Wisteria floribunda* lectin (WFA), Soybean agglutinin (SBA) and *Vicia villosa* agglutinin (VVA)], and (3) N-acetylglucosamine residues [Wheat germ agglutinin (WGA)]. Representative images and a summary of the major changes are shown in Fig. 3.

Wandering L3 control and *dGALT*-null larvae were probed with each lectin label (green in Fig. 3) and compared to anti-Fasciclin-II (FasII; red in Fig. 3) as a synaptic marker for the NMJ terminal. FasII intensity remained unchanged across genotypes in all studies (data not shown). In sharp contrast, the ECL lectin, binding non-reducing terminal galactose, strongly labeled the NMJ synaptomatrix in controls (*n*=8), but labeling was dramatically decreased in mutants (*n*=9, *P*=0.0025; Fig. 3A,A′). Similarly, WFA lectin, binding to terminal N-acetylgalactosamine residues, strongly labeled control NMJs, but labeling was also strikingly reduced in *dGALT*-null NMJs (*n*=12, *P*=0.0016; Fig. 3B,B′). Moreover, PNA and VVA lectins, binding galactosyl(β-1,3)N-acetylgalactosamine (T-antigen) and N-acetylgalactosamine terminal glycans, respectively, both labeled wild-type NMJs, and showed a more modest but significant reduction in *dGALT* nulls (PNA, *n*=8, 16% reduction, *P*=0.0496; VVA, *n*=14, 22% reduction, *P*=0.0497; data not shown). We also assayed the NMJ with SBA and WGA lectins, which bind to terminal N-acetylgalactosamine (α and β) and N-acetylglucosamine, respectively, but found no differences between genotypes (data not shown). Finally, we probed *dGALT*-null NMJs with the commonly employed synaptic marker anti-HRP (Jan and Jan, 1982) to test for α1,3-fucosylation, because glycan fucosylation defects are reported in untreated galactosemia (Sturiale et al., 2005). We found that *dGALT*-null NMJs show a striking loss of the HRP glycan (control: 1.0±0.07, *n*=9; *dGALT^ΔAP2^*: 0.58±0.10, *n*=8; *P*=0.005; Fig. 3C,C′). The results indicate profound differences in the NMJ glycosylated synaptomatrix in this CG disease model.

Because control animals fed galactose replicate *dGALT* phenotypes, we next explored whether galactose feeding in wild-type larvae alters the NMJ glycosylation composition, by assaying WFA lectin expression with and without dietary intervention. WFA lectin strongly labeled unsupplemented control NMJs (normalized 1.0±0.05, *n*=8) but, similar to *dGALT* mutants, was strikingly reduced in larvae raised on a high-galactose diet (0.78±0.07; *n*=7, *P*=0.02). To further test the role of *dGALT* as a modifier of NMJ glycosylation state, transgenic *hGALT* rescue animals were compared with two control groups (wild type, and driver-alone in the *dGALT^ΔAP2^* background) by assaying WFA expression. The loss of WFA lectin signal in *dGALT* nulls (0.72±0.07; *n*=18) was completely rescued with transgenic expression of *hGALT* (1.08±0.12; *n*=7) to a level undistinguishable from wild-type controls (1.0±0.07; *n*=19). Taken together, these results reveal striking alterations in NMJ glycan composition in the absence of *dGALT* activity, specifically including reductions in galactosyl and N-acetylgalactosamine residues, and fucosylated HRP glycans. Because the NMJ synaptomatrix carbohydrate environment limits synaptic morphogenesis via the modulation of trans-synaptic signaling of the Wnt Wg, the pathway that drives morphological synaptogenesis (Dani et al., 2012; Parkinson et al., 2013), we next characterized components of this signaling pathway at the NMJ.

### *dGALT* mutants display changes in Wnt trans-synaptic signaling pathway components

**Fig. 4.**
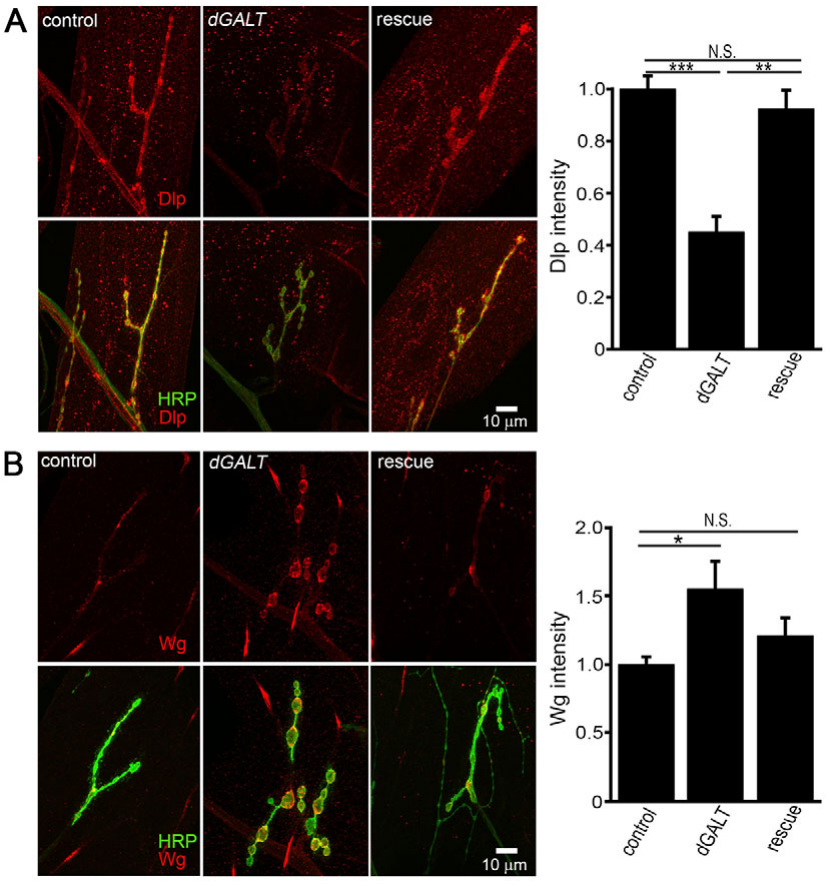
**Null *dGALT* NMJs exhibit an altered Wnt trans-synaptic signaling pathway.** (A) Representative NMJs imaged with anti-horseradish-peroxidase (HRP; green) and anti-Dally-like-protein (Dlp; red) in genetic control (*dGALT^C2^*), *dGALT*-null (*dGALT^ΔAP2^*) and rescue (*dGALT^ΔAP2^; UH1-Gal4/UAS-hGALT*) larvae. Right: quantification of Dlp intensity normalized to control (*dGALT^C2^*). (B) Representative NMJs probed with anti-HRP (green) and anti-Wingless (Wg; red) in the same genotypes. Right: quantification of Wg intensity normalized to control. Sample size: ≥ten NMJs. Error bars show s.e.m. with significance indicated: *P<0.05, **P<0.01, ***P<0.001.

Glycan mechanisms within the heavily glycosylated *Drosophila* NMJ synaptomatrix have been repeatedly shown to regulate synaptic morphogenesis (Dani and Broadie, 2012). In particular, glycans modulate the trans-synaptic signaling that drives synaptogenesis (Dani et al., 2012; Friedman et al., 2013; Parkinson et al., 2013). More specifically, the HSPG Dlp has been identified as a mediator of synapse formation (Johnson et al., 2006; Van Vactor et al., 2006). At the *Drosophila* NMJ, Dlp acts as a co-receptor of the Wnt protein Wg to limit extracellular Wg availability and presentation, regulating NMJ development (Dani et al., 2012; Friedman et al., 2013). Importantly, this mechanism is linked to the Leloir pathway via UDP-glc dehydrogenase (Fig. 1A), whose loss reduces Dlp biosynthesis (Binari et al., 1997; Toyoda et al., 2000). We therefore hypothesized that loss of *dGALT* would alter Dlp expression to misregulate Wg ligand abundance, with the change in Wg trans-synaptic signaling providing a mechanistic basis for NMJ synaptogenesis defects in the CG disease model. To test this hypothesis, we probed NMJs from wandering L3 larvae with anti-Dlp and -Wg, using anti-HRP as the synaptic marker. Data and representative images are shown in Fig. 4.

In controls, Dlp expression overlapped that of the HRP synaptic marker throughout most of the NMJ, extending slightly beyond the HRP boundaries (Fig. 4A). Compared with the strong Dlp expression in controls (*dGALT^C2^*, *n*=20), *dGALT*-null NMJs (*dGALT^ΔAP2^*, *n*=5) showed a >50% reduction in Dlp levels (normalized control: 1.0±0.10; *dGALT^ΔAP2^*: 0.45±0.06; *P*<0.0001; Fig. 4A, right). Transgenic *hGALT* expression rescued Dlp to wild-type levels (0.92±0.07, *n*=18), with no significant difference remaining between rescue animals and the genetic control. We next tested Wg ligand levels in the same genotypes (Fig. 4B). In controls, Wg is expressed at low, variable levels surrounding NMJ boutons, with dynamic, bouton-specific regions of high Wg expression (Dani et al., 2012; Friedman et al., 2013; Parkinson et al., 2013). Conversely, *dGALT*-null NMJs displayed a >50% increase in Wg ligand levels (control: 1.0±0.07, *n*=23; *dGALT^ΔAP2^*: 1.55±0.20, *n*=23), a significant elevation compared with the genetic control (*P*<0.05; Fig. 4B, right). Transgenic expression of *hGALT* decreased Wg ligand towards wild-type levels (1.21±0.12, *n*=10), with no significant difference to controls. Taken together, these results reveal changes in Wg co-receptor and ligand levels, with the loss of the HSPG Dlp co-receptor providing a well-established mechanism to explain the elevation of Wg ligand in the NMJ synaptomatrix.

### Co-removal of *dGALK* prevents the movement and NMJ defects in *dGALT* nulls

**Fig. 5.**
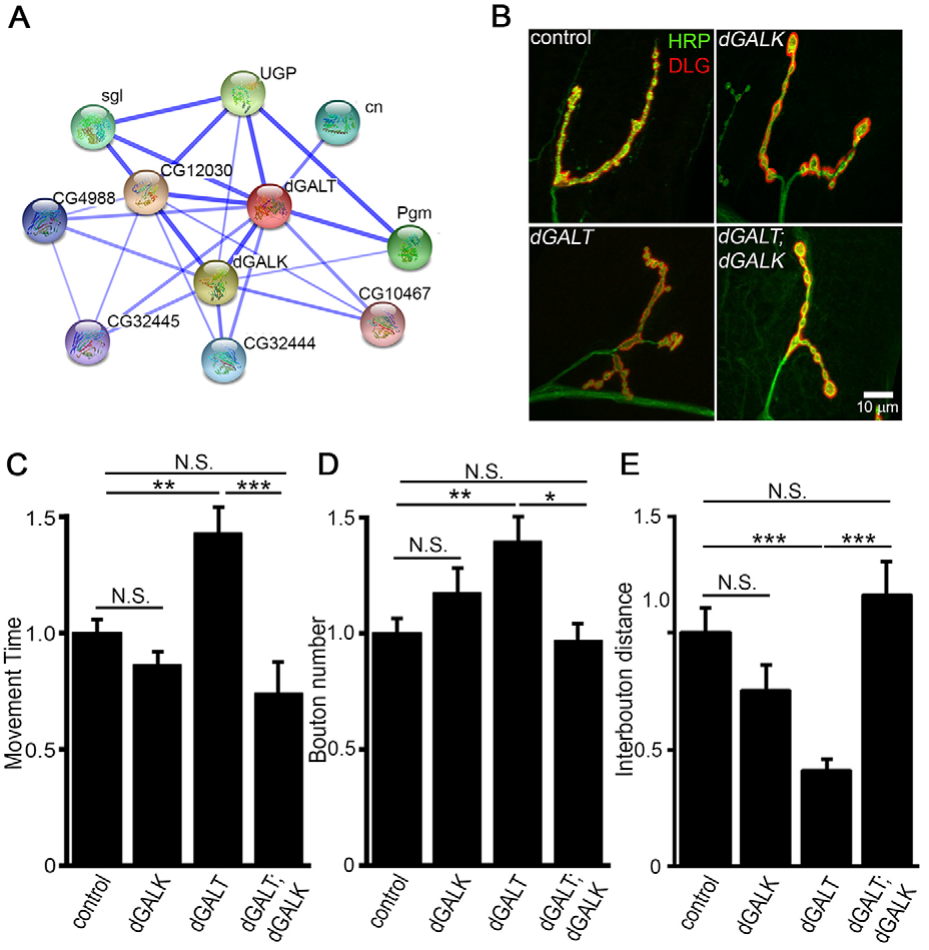
**Co-removal of *dGALK* prevents *dGALT* movement and NMJ structural defects.** (A) Protein-protein interaction network for dGALT generated with the Search Tool for Retrieval of Interacting Genes (STRING). Line thickness represents the strength of predicted interactions. (B) Representative NMJs imaged with anti-horseradish-peroxidase (HRP; green) and anti-Discs-large (DLG; red) in control (*dGALT^C2^*), *dGALK*-null (*dGALK^ΔEXC9^*), *dGALT*-null (*dGALT^ΔAP2^*) and double-null mutant (*dGALT^ΔAP2^; dGALK^ΔEXC9^*) larvae. (C) Normalized time required for wandering L3 to rollover for all four genotypes. (D) Quantification of NMJ bouton number and (E) inter-bouton distance, normalized to control. Sample size: ≥seven animals for each genotype. Error bars show s.e.m. with significance indicated: *P<0.05, **P<0.01, ***P<0.001, not significant (*P*>0.05, N.S.).

**Fig. 6.**
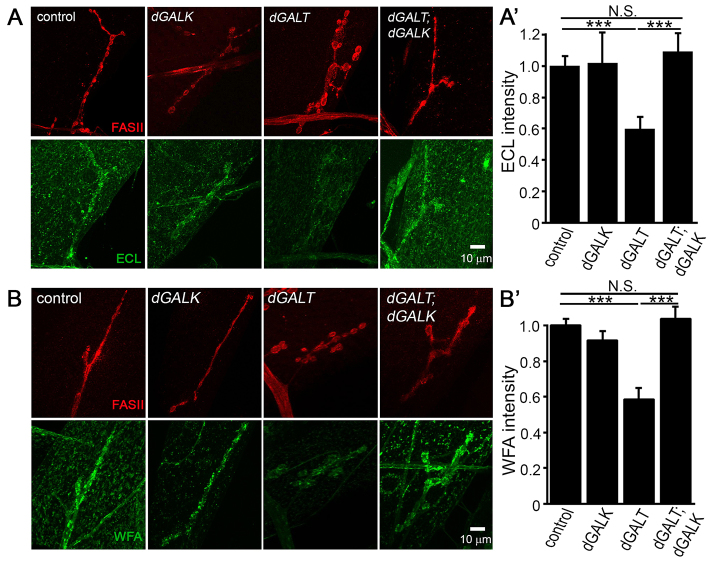
**Co-removal of *dGALK* prevents the loss of galactosylation in the *dGALT*-null synaptomatrix.** Representative NMJs imaged with anti-Fasciclin-II (FASII; red) and (A) *Erythrina cristagalli* lectin (ECL; green) or (B) *Wisteria floribunda* agglutinin (WFA; green) in control (*dGALT^C2^*), *dGALK*-null (*dGALK^ΔEXC9^*), *dGALT*-null (*dGALT^ΔAP2^*) and double-null (*dGALT^ΔAP2^; dGALK^ΔEXC9^*) larvae. Normalized quantification of ECL (A′) and WFA (B′) intensities. Sample size: ≥six NMJs. Error bars show s.e.m. with significance indicated: ***P<0.001, not significant (*P*>0.05, N.S.).

To explore a systems-level understanding of GALT-dependent functions at the synapse, we used the Search Tool for Retrieval of Interacting Genes (STRING) (Szklarczyk et al., 2011) database to identify candidate dGALT-interacting gene products (Fig. 5A). We identified dGALK as a highly associated dGALT interactor. This is not surprising, because dGALK is directly upstream of dGALT in the Leloir pathway (Fig. 1A). Moreover, it is commonly conjectured that gal-1-P accumulation is central to the symptoms of individuals with CG (Gitzelmann, 1995; Webb et al., 2003; Tang et al., 2010; Cangemi et al., 2012), suggesting that *dGALK* co-removal should ameliorate *dGALT*-null phenotypes in our CG disease model. To test this hypothesis, we generated *dGALT; dGALK* double-null mutants, and then retested all of the behavioral, NMJ architecture and glycosylated synaptomatrix phenotypes described above. A summary of these analyses is shown in Figs 5 and 6.

First, co-removal of *dGALK* fully corrected the behavioral movement defects of *dGALT* nulls (Fig. 5C). Null *dGALT* larvae again showed a slowed movement time (and movement was uncoordinated) compared with genetic controls (control: 17.2±1.0 s, *n*=37; *dGALT^ΔAP2^*: 24.3±2.1 s, *n*=32), which was fully restored to the wild-type condition in double mutants (*dGALT; dGALK*: 12.2±2.2 s, *n*=20), a highly significant improvement (*P*<0.001; Fig. 5C). Likewise, the NMJ structural overelaboration characterizing *dGALT*-deficient larvae was prevented by co-removing *dGALK* (Fig. 5B). Compared with the elevated synaptic bouton number in single nulls (homozygous *dGALT^ΔAP2^*: 34.5±2.5, *n*=14), bouton number was significantly decreased in double mutants (*dGALT; dGALK*: 23.5±1.5, *n*=8, *P*<0.05) and was indistinguishable from wild-type animals (control 24.5±1.7 boutons, *n*=15; Fig. 5D). Similarly, *dGALK* co-removal in *dGALT* mutants rescued the reduced interbouton distance characterizing the single mutant (*dGALT; dGALK*: 0.96±0.08 μm, *n*=8; *dGALT* 0.33±0.04 μm, *n*=13, *P*<0.001; Fig. 5E). Removal of *dGALK* alone did not impact coordinated movement time (control: 15.3±1.5 s, *n*=21; *dGALK*: 13.2±0.8 s, *n*=22), NMJ bouton number (control: 20.7±2.3, *n*=6; *dGALK*: 24.3±2.1, *n*=7) or interbouton distance (control: 1.05±0.2 μm; *dGALK*: 0.75±0.1 μm), which were all indistinguishable from controls.

To test the hypothesis that *dGALK* co-removal would prevent the losses in glycosylation of the NMJ synaptomatrix observed in *dGALT* nulls, we next probed double mutants with lectins for the three glycans with the largest changes in levels observed in the single mutants (Fig. 3): ECL lectin for terminal galactosyl residues, WFA lectin for N-acetylgalactosamine residues and anti-HRP for fucosylated HRP epitopes (Fig. 6). The losses of ECL and WFA lectin labeling in *dGALT* nulls completely disappeared with *dGALK* co-removal (Fig. 6A,B). ECL lectin labeling was once again reduced by ~50% in *dGALT* nulls (*P*<0.001), but *dGALK* co-removal restored ECL labeling to control levels (*n*=19; Fig. 6A′). Loss of *dGALT* activity similarly decreased WFA labeling by ~50% (*n*=17) compared with controls (*n*=23, *P*<0.001), but WFA labeling was again fully restored to control levels in double mutants (*n*=10; Fig. 6B′). Loss of GALK alone impacted neither ECL nor WFA labeling (*n*=6 for both). In contrast, the reduced HRP signal observed in *dGALT*-null NMJs was unaffected by eliminating *dGALK* in parallel: in the absence of *dGALT* (0.74±0.06, *n*=22), there was a highly significant reduction in HRP labeling in mutants compared with controls (1.0±0.03, *n*=36, *P*<0.001), but there was no significant improvement with *dGALK* co-removal (0.81±0.05, *n*=25). Thus, the double mutant selectively restores synaptomatrix glycosylation state, but does not completely reverse NMJ glycan changes.

**Fig. 7.**
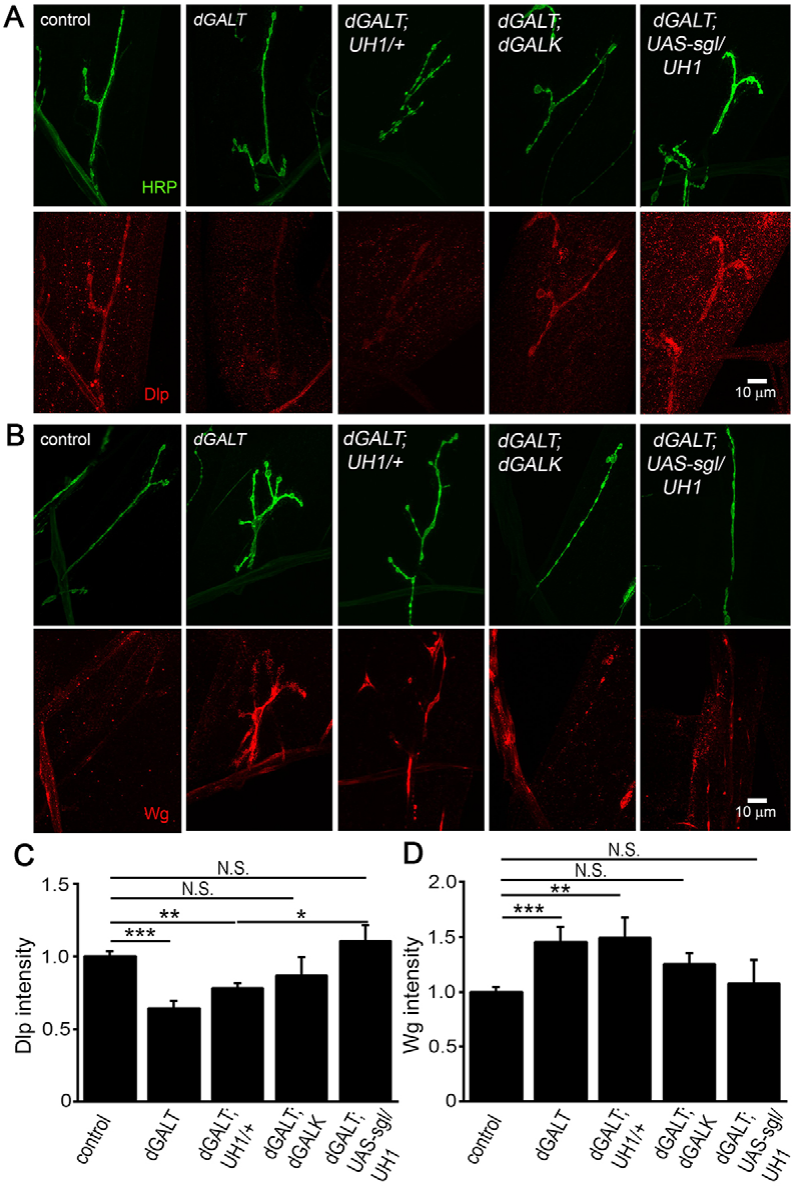
**Restoration of Wnt trans-synaptic signaling components in *dGALT*-null NMJs.** Representative NMJs imaged with anti-horseradish peroxidase (HRP; green) and anti-Dally-like-protein (Dlp; red) (A) or anti-Wingless (Wg; red) (B) in control (*dGALT^C2^*), *dGALT* null (*dGALT^ΔAP2^*), *dGALT* null with driver alone (*dGALT^ΔAP2^*; *UH1/+)*, *dGALT; dGALK* double null (d*GALT^ΔAP2^; dGALK^ΔEXC9^*) and *dGALT* null with *sgl* overexpression (*dGALT^ΔAP2^; UH1-Gal4/UAS-hGALT*). Quantification of Dlp (C) and Wg (D) intensity normalized to genetic control (*dGALT^C2^*). Sample size: ≥eight NMJs (Dlp) and ≥five NMJs (Wg) per genotype. Error bars show s.e.m. with significance indicated: *P<0.05, **P<0.01, ***P<0.001.

In light of such glycosylation state changes, we next tested whether *dGALK* co-removal also restores Dlp and Wg levels (Fig. 7). Normalized to controls, reduced Dlp levels characterizing *dGALT* nulls (0.64±0.05, *n*≥8, *P*<0.001) were significantly elevated towards wild-type values in double mutants (0.87±0.13, *n*≥9, *P*<0.01) and were no longer significantly different from controls (Fig. 7A,C). Likewise, elevated Wg levels in *dGALT*-null NMJs (1.45±0.14, *n*≥8, *P*<0.001) were reduced towards wild-type levels in *dGALT; dGALK* double nulls (1.25±0.10, *n*≥9), and were no longer significantly different from controls (Fig. 7B,D). Thus, the reduced levels of Dlp co-receptor and increased levels of Wg ligand at *dGALT*-null NMJs were significantly corrected by co-removal of *dGALK* activity. Taken together, these results demonstrate that *dGALK* is a key genetic modifier in the *Drosophila* CG disease model, raising the intriguing possibility that inhibition of GALK activity in patients might similarly modulate CG neurological symptoms. Because *sgl* is essential for Dlp biosynthesis (Häcker et al., 1997), we next tested whether increasing Dlp biosynthesis via *sgl* overexpression could similarly prevent defective NMJ synaptogenesis and impaired movement.

### Overexpression of *sgl* prevents the movement and NMJ defects in *dGALT* nulls

**Fig. 8.**
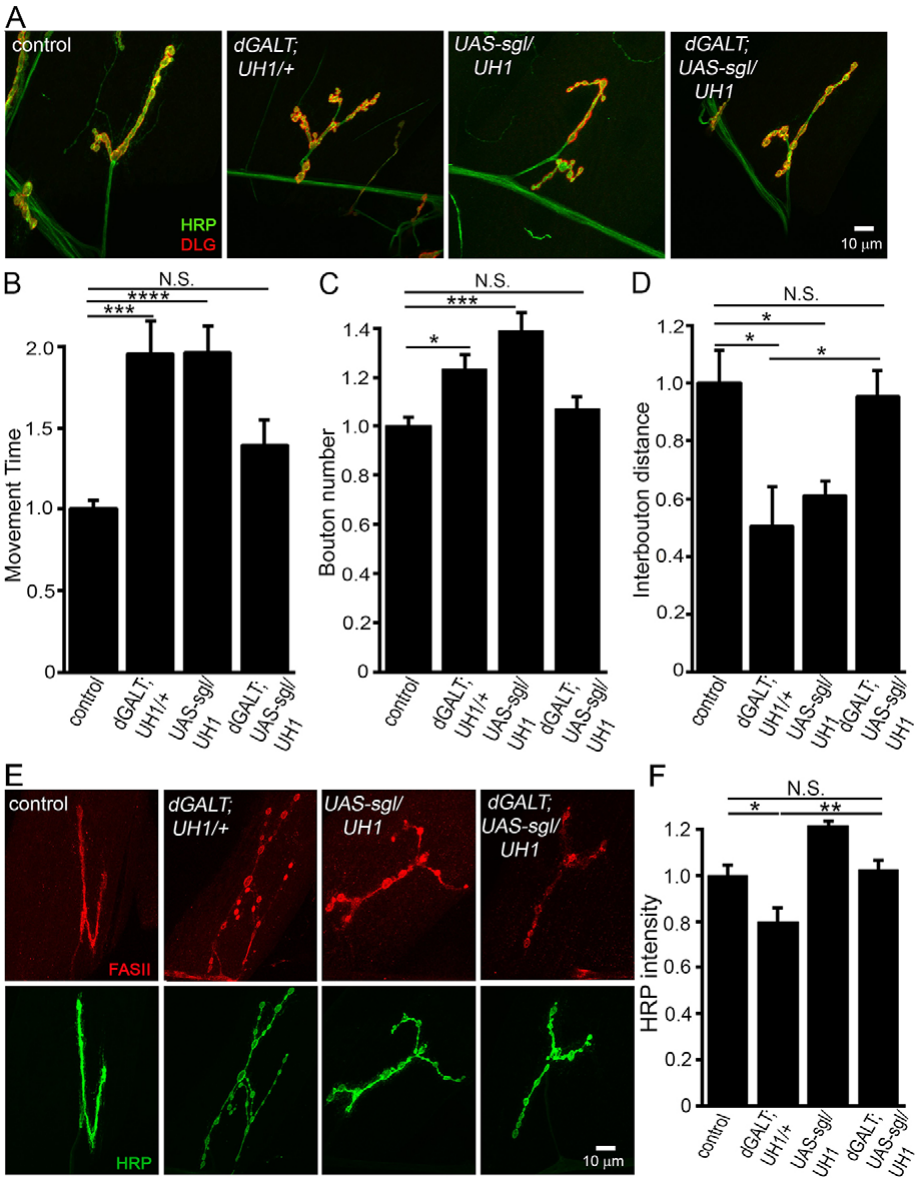
***sgl* overexpression prevents *dGALT* motor, NMJ and HRP glycosylation defects.** (A) Representative NMJs imaged with anti-horseradish-peroxidase (HRP; green) and anti-Discs-large (DLG; red) for control (*dGALT^C2^*), *dGALT* null with driver alone (*dGALT^ΔAP2^; UH1/+), sgl* overexpression (*UAS-sgl/UH1-Gal4*) and *dGALT* null with *sgl* overexpression (*dGALT; UAS-sgl/UH1-Gal4*). (B) Normalized time required for the wandering L3 to rollover for all four genotypes. (C) Quantification of synaptic bouton number and (D) inter-bouton spacing distance. (E) Sample NMJs imaged with anti-Fasciclin-II (FASII; red) and anti-HRP (green). (F) Normalized quantification of HRP intensity. Sample size: ≥seven NMJs. Error bars show s.e.m. with significance indicated: *P<0.05, **P<0.01, ***P<0.001, ****P<0.0001, not significant (*P*>0.05, N.S.).

Our STRING analysis (Szklarczyk et al., 2011) revealed the *Drosophila sgl* gene product to be strongly associated with dGALT (Fig. 5A). The *sgl* gene encodes UDP-glc dehydrogenase, which makes UDP-glucuronate from UDP-glc (Fig. 1A), an activity that is essential for the biosynthesis of proteoglycans (Häcker et al., 1997). Importantly, *sgl* mutants have a compromised production of the HSPG co-receptor Dlp (Haerry et al., 1997), which in turn regulates the Wg ligand driving *Drosophila* NMJ synaptogenesis (Dani et al., 2012), as discussed above (see Fig. 4). We therefore hypothesized that overexpressing *sgl* should prevent *dGALT* mutant phenotypes by antagonistically increasing Dlp levels, thus acting to limit Wg abundance to properly control NMJ morphogenesis and coordinated movement behavior. To test this hypothesis, we made transgenic animals overexpressing *sgl* (Monnier et al., 2002) in the *dGALT*-null background (*dGALT^ΔAP2^*/*dGALT^ΔAP2^*; *P{Mae-UAS.6.11}sgl^UY771^*/*UH1-Gal4*) to assay the movement behavior, NMJ architecture, glycosylated synaptomatrix and Wnt pathway phenotypes described above. A summary of these data is shown in Fig. 8.

*UH1-Gal4* driven UAS-*sgl* overexpression in *dGALT* nulls strongly restored coordinated movement (Fig. 8B). Loss of *dGALT* activity nearly doubled rollover time compared with matched controls (control: 13.7±0.9 s, *n*=54; *dGALT^ΔAP2^; UH1-Gal4*/+: 26.1±2.3 s, *n*=39, *P*<0.0001), but *sgl* overexpression strongly decreased movement time, eliminating any significant difference with controls [*dGALT; UAS-sgl/UH1-Gal4*: 18.8±2 s, *n*=32; not significant (N.S.) compared with control; Fig. 8B]. Moreover, similar to single *dGALT* mutants, *sgl* overexpression alone delayed movement time (*UH1-Gal4*/+: 13.9±1.4 s, *n*=20; *UAS-sgl^UY771^*/*UH1-Gal4*: 27.4±2.2 s, *n*=17), compared with controls (*P*<0.0001; Fig. 8B). For NMJ architecture, *dGALT* nulls with *sgl* overexpression lacked the overelaboration of single-mutant conditions (Fig. 8A,C). Compared with the supernumerary boutons characterizing *dGALT*-null NMJs (control: 26.9±1.3 boutons, *n*=16; *dGALT; UH1-Gal4/+*: 36±1.7 boutons, *n*=7), concomitant overexpression of *sgl* significantly decreased bouton number (*dGALT; UAS-sgl/UH1-Gal4*: 28.9±1.5 boutons, *n*=24; Fig. 8A,C). Synaptic bouton number increased >30% with *sgl* overexpression, compared with matched control (*UH1-Gal4*/+: 24.5±2.0, *n*=12; *UAS-sgl/UH1*: 31.6±1.1, *n*=18; *P*=0.0009; Fig. 8C). The overexpression of *sgl* in *dGALT* nulls similarly restored interbouton distance to wild-type levels (control: 0.86±0.10 μm; *dGALT*: 0.46±0.12 μm; *dGALT; UAS-sgl/UH1-Gal4*: 0.83±0.09; Fig. 8D).

To test whether *sgl* overexpression modifies glycosylated synaptomatrix composition, antibody and lectin glycan labeling were assayed. Whereas *sgl* overexpression alone did not impact levels of HRP compared with driver-alone controls (*UAS-sgl/UH1-Gal4*: 1.21±0.02, *n*=7; *UH1-Gal4/+*: 1±0.09, *n*=6; Fig. 8E), loss of fucosylated HRP glycans in *dGALT* nulls was fully prevented by *sgl* overexpression (*n*=14; Fig. 8E), a highly significant improvement compared with single mutants (*dGALT; UH1-Gal4/+*: *n*=10, *P*<0.01; Fig. 8F). Conversely, the significantly reduced WFA labeling in *dGALT* mutants remained unaffected in double mutants. In *dGALT* nulls, there was a significant reduction in WFA lectin (control: 1±0.09, *n*=12; *dGALT; UH1-Gal4/+*: 0.70±0.07, *n*=8; *P*<0.05), which failed to improve with *sgl* overexpression (0.71±0.1, *n*=9). Note that the restoration of glycosylated synaptomatrix composition in *dGALT* nulls was exactly opposite for *dGALK*-null and *sgl*-overexpression double-mutant conditions (Figs 6 and 8). Despite these differences, *sgl* overexpression in *dGALT* nulls restored both loss of the Wg co-receptor Dlp (normalized control: 1.0±0.04, *n*=25; *dGALT; UH1-Gal4/+*: 0.78±0.04, *n*=14, *P*<0.01; *dGALT; UAS-sgl/UH1-Gal4*: 1.11±0.11, N.S. compared with control; Fig. 7A,C) and Wg ligand elevation (control: 1.0±0.05, *n*=31; *dGALT; UH1-Gal4/+*: 1.49±0.18, *n*=17; *P*<0.01; *dGALT; UAS-sgl/UH1-Gal4*: 1.07±0.22, N.S.; Fig. 7B,D). Taken together, these findings show that concomitant overexpression of *sgl* is a strong positive modifier of outcome in this CG model.

## DISCUSSION

Loss of motor coordination has long been reported as one of the most frequent CG symptoms (Böhles et al., 1986; Waggoner et al., 1990; Schweitzer et al., 1993; Kaufman et al., 1995; Hughes et al., 2009). Consistently, the *Drosophila* CG disease model showed reduced activity and impaired movement coordination, which were fully rescued by transgenic expression of human *GALT*, demonstrating full functional conservation. The mechanistic basis of CG movement defects remains unknown after 50 years of study (Bosch, 2006). However, our recent *Drosophila* screen of glycogenes identified a role of *dGALT* in NMJ morphological synaptogenesis (Dani et al., 2012). In light of previous reports in rodent (Audouard et al., 2012) and *Drosophila* (Feiguin et al., 2009) providing compelling evidence associating deficient movement behaviors with NMJ structural abnormalities, we set forth to characterize NMJ synaptic architecture under conditions of complete and targeted loss of *dGALT* activity. These studies reveal elevated synaptic growth and structural overelaboration, albeit without a change in basal NMJ transmission strength. These findings are consistent with previous studies showing that rollover movement defects occur independently of synaptic transmission defects (Bodily et al., 2001), and suggest that defective NMJ architecture impairs the muscle control that is required to coordinate the sequence of motor outputs to optimally produce a bilateral coordinated movement (twist-and-roll behavior). These NMJ structural defects are also consistent with our earlier work showing that loss of specific N-glycans (Dani et al., 2012), or the whole cassette of complex/branched N-glycans (Parkinson et al., 2013), removes constraints on NMJ growth and structural elaboration. We conclude that glycans play a primarily inhibitory role in modulating synapse morphogenesis.

Major controversies surround the benefits of a galactose-restricted diet in the CG disease state (Jumbo-Lucioni et al., 2012), particularly in alleviating chronic neurological symptoms (Bosch, 2006). We therefore tested impacts of galactose in the diet on movement and NMJ structure in our *Drosophila* model. A high-galactose diet did not impact either the impaired movement or overelaborated NMJ architecture of *dGALT* mutants, consistent with earlier reports that *dGALT* long-term phenotypes are independent of dietary galactose (Ryan et al., 2012). Strikingly, however, wild-type animals fed a high-galactose diet phenocopied many *dGALT*-mutant phenotypes, displaying slowed coordinated movement and grossly overelaborated NMJs with supernumerary boutons over decreased interbouton distances. Previous studies on experimental models of galactosemia in genetically wild-type animals (Cui et al., 2006; Wei et al., 2005; Long et al., 2007) demonstrate that exposure to high levels of galactose leads to neurodegeneration and cognitive disability, some of the chronic complications reported in CG. Increased dietary galactose decreases the UDP-glc:UDP-gal ratio (Gibson et al., 1995; Gibson et al., 1996). Because glycosylation depends upon UDP-sugar availability, NMJ architectural defects in both wild type overfed galactose and *dGALT* nulls might result from disrupted UDP-sugar balance and consequently impaired glycosylation. *Drosophila* larvae have been shown to accumulate significantly higher levels of gal-1-P on a high-galactose diet (Kushner et al., 2010), which has been shown to directly impact UDP-sugar balance (Lai et al., 2003). In contrast to the *Drosophila* CG model, the viability of mice deficient in GALT activity and reared on a high-galactose diet is reportedly unaffected, despite the accumulation of very high gal-1-P levels (Leslie et al., 1996; Ning et al., 2001). A theory to explain this discrepancy is that very low levels of aldose reductase activity in mice prevent the accumulation of galactitol, a toxic galactose intermediate. A newly established *GALT*-null mouse model (Tang et al., 2014) reveals reduced viability and abnormal cellular changes in the brain as a result of galactose exposure, but fails to inform whether such changes persist under galactose restriction. Our findings in galactose-fed *dGALK* mutants suggest that gal-1-P accumulation is a primary determinant of the behavioral deficits characterizing *dGALT* mutants, and wild-type animals fed a high galactose diet.

Glycan-binding lectins have long been used to define the extracellular glycan landscape at the NMJ synapse (Ohtsubo and Marth, 2006; Martin and Freeze, 2003; Scott et al., 1988). Null *dGALT* NMJs displayed striking glycosylation defects, including substantial reductions in galactosyl, N-acetylgalactosamine and fucosylated HRP moieties. Differences between lectin probes arise from different binding specificity. ECL and PNA lectins both bind terminal galactose, whereas PNA binds preferentially Gal(β-1,3)-GalNAc and ECL binds Gal(β1,4)-GlcNAc. Similarly, WFA recognizes terminating N-acetylgalactosamine (α/β-linked to galactose) and VVA binds preferentially to Tn-antigen (i.e. a single α-GalNAc residue linked to serine or threonine). Earlier studies examining the molecular basis of neural anti-HRP staining in *Drosophila* have demonstrated specific recognition of core α1,3-fucosylated glycoproteins (Fabini et al., 2001). Galactose-containing glycans have key synaptogenesis roles (Tai and Zipser, 1999), and glycosylation losses in rodents (Lowe and Marth, 2003) and specific loss of HRP epitopes in *Drosophila* (Baas et al., 2011) have both been independently linked to motility abnormalities. However, although other CDG models also display evidence of overelaborated motor neuron architectures (Cline et al., 2012), the correlation with loss of HRP epitopes is less clear. Two previous studies have shown a reduction of HRP expression with underelaborated *Drosophila* NMJ structure (Rendić et al., 2010; Baas et al., 2011), in contrast to our results, whereas a recent study from our lab demonstrated HRP loss accompanied by increased synaptic growth and structural overelaboration (Parkinson et al., 2013), as in the current study. Such complicated carbohydrate-mediated tuning of NMJ synaptogenesis might explain the correction of the synaptic architectural overelaboration in *dGALT* nulls with *dGALK* co-removal or *sgl* overexpression, despite the differential and complementary correction of synaptomatrix glycosylation losses.

NMJ synaptogenesis requires bidirectional trans-synaptic signaling via secreted glycoprotein ligands (Carbonetto and Lindenbaum, 1995; Dani et al., 2012; Collins and DiAntonio, 2007). Such signals must necessarily traverse the heavily glycosylated synaptomatrix, and we have shown that the glycan state of this extracellular environment is crucially important for enabling and shaping trans-synaptic signaling during NMJ synaptogenesis (Rohrbough and Broadie, 2010; Rushton et al., 2012; Parkinson et al., 2013). This synaptomatrix glycosylation state is strongly compromised in our CG disease model, suggesting that signaling should be similarly altered. Consistently, *dGALT* mutants exhibited elevated Wnt Wg ligand, the best-characterized trans-synaptic signal at the *Drosophila* NMJ (Kamimura et al., 2013; Miech et al., 2008) The HSPG Dlp acts as a Wg co-receptor, regulating both extracellular distribution and signaling (Han et al., 2005; Kirkpatrick et al., 2004), and we have recently established that Dlp limits Wg trans-synaptic signaling at the *Drosophila* NMJ (Dani et al., 2012; Friedman et al., 2013). Consistently, *dGALT* nulls exhibited a sharp decrease in Dlp levels, providing a mechanism for Wg overexpression. Wg overexpression in turn is well known to increase NMJ bouton formation (Packard et al., 2002; Ataman et al., 2008), providing a mechanism to explain the supernumerary boutons characterizing our CG disease model. Importantly, defects in trans-synaptic Wg co-receptor and ligand levels are rescued with transgenic expression of *hGALT*, showing functional conservation.

We identify two key *dGALT* genetic interactors: (1) *dGALK*, upstream in the Leloir pathway, and (2) *sgl*, a downstream UDP-glc dehydrogenase. GALK catalyzes galactose phosphorylation to gal-1-P, whose accumulation is linked to CG symptoms (Pesce and Bodourian, 1982; Cangemi et al., 2012), although recent studies have challenged this conclusion (Jumbo-Lucioni et al., 2013; Jumbo-Lucioni et al., 2014). The current work shows that *dGALK* co-removal in *dGALT* nulls restores normal movement behavior, NMJ architecture and synaptomatrix galactosylation. Evidence in support of this from individuals with CG shows that pathological accumulation of gal-1-P directly reduces both UDP-glc and UDP-gal bioavailability in GALT deficiency (Lai et al., 2003). Similarly, earlier studies have shown that elimination of GALK activity via GALK pharmacological inhibitors in CG patient cells (Tang et al., 2010) or *GALK* deletion in yeast (De-Souza et al., 2014) prevents gal-1-P accumulation, relieves galactose toxicity and restores UDP-sugar balance (Lai et al., 2003).

Sgl is required for the synthesis of the HSPG co-receptor Dlp (Toyoda et al., 2000; Haerry et al., 1997), which modulates Wg signaling, as described above. Consistently, *sgl* mutants recapitulate *Wg* phenotypes (Superina et al., 2014; Haerry et al., 1997). This established interaction strongly supports findings here showing that *sgl* is a strong genetic modifier in our CG disease model. Moreover, *sgl* overexpression by itself phenocopies loss of *dGALT*, and *sgl* gain-of-function in *dGALT* mutants restores NMJ architecture, Wg trans-synaptic signaling components and motor behavior output. In wild type, increased UDP-glc dehydrogenase activity can augment hyaluronan production, glycosaminoglycan release and extracellular matrix elaboration (Clarkin et al., 2011), and thus impact glycosylated synaptomatrix composition to account for negative outcomes of *sgl* overexpression. Conversely, overproduction of extracellular glycans is predicted to be beneficial under conditions of a depleted glycosylated synaptomatrix, as occurs in *dGALT* nulls. Furthermore, Dlp and Wg levels are both strongly corrected toward control values by *dGALK* co-removal and *sgl* overexpression genetic interventions. These findings strengthen the argument that the Wnt signaling pathway plays a crucial role in the pathogenesis of neurological complications in CG. Taken together, these results suggest that both GALK inhibition and UDP-glc dehydrogenase activation might combat neurological symptoms in individuals with CG.

In conclusion, the results presented here are the first to reveal NMJ glycosylation losses, synaptic architecture defects and concomitant changes in trans-synaptic Wnt co-receptor and ligand levels in a CG disease model. These findings suggest a model in which loss of GALT activity triggers gal-1-P accumulation, which subsequently limits UDP-sugar bioavailability. In this model, UDP-sugar deficits trigger changes in NMJ glycosylated synaptomatrix composition, including levels of GPI-anchored HSPG Dlp, an essential co-receptor for a Wnt ligand during NMJ synaptogenesis. Loss of HSPG regulation results in failure to control Wg levels, causing differential trans-synaptic Wnt signaling to promote excess growth and overelaborated architectural complexity. Co-removal of *dGALK*, and *sgl* overexpression, both are strong genetic modifiers of outcome in this CG model. We propose that similar defects underlie well-characterized glycosylation defects and movement disorders in the human CG disease state, and might account for neurological pathogenesis characterizing a wide array of related CDG disease states, which will be the subject of our future investigations.

## MATERIALS AND METHODS

### *Drosophila* genetics

Fig. 1A shows the Leloir pathway, listing enzyme names and *Drosophila* CG numbers of genes targeted in this study. A *dGALT* imprecise-excision null, *dGALT^ΔAP2^*, with undetectable enzyme activity, and a precise-excision control, *dGALT^C2^*, with normal enzyme activity, were previously described (Kushner et al., 2010). The genomic deficiency *Df(2L)BSC187* crossed to *dGALT^ΔAP2^* was used as a second heterozygous-null condition. Ubiquitous (*UH1*) and tissue-specific (neuronal *elav* and muscle *24B*) Gal4 drivers were used to drive *dGALT*-RNAi (*v10025; P{KK102974}VIE-260B*, Vienna *Drosophila* RNAi Center). Gal4 drivers alone (*Gal4/+*) crossed to *w^1118^* were genetic controls. A *dGALK* imprecise-excision null, *dGALK^ΔEXC9^*, with undetectable enzyme activity, was generated by mobilizing a P-element insertion (*EY03791*) in the 5′-UTR of *CG5068*. UAS-*sgl^UY771^* was used to overexpress *sgl* (Monnier et al., 2002). *dGALT*, *dGALK* and *sgl* alleles were combined using standard genetic techniques to generate double mutants. Single and double mutants were reared at 25°C on standard molasses-based food. To test effects of a high-galactose diet, food was supplemented with 200 mM galactose.

### Behavioral assays

All behavioral experiments were carried out on at least eight individual animals for each genotype. Locomotor activity was assayed in adult males using the *Drosophila* Activity Monitoring (DAM) system (Trikinetics, Waltham, MA). Starting 1 day after eclosion, animals were entrained to a 12-hour light/dark cycle for 2 days before data recording, with activity counts per hour recorded for 3 days (Chiu et al., 2010). Coordinated larval movement was assayed in male wandering third instars (L3) using the rollover assay described previously (Bodily et al., 2001; Pan et al., 2008). Animals were placed individually on a room temperature (RT) 1% agar plate and allowed to acclimate for 2 minutes. The L3 animal was then rolled to an inverted position as defined by the ventral midline. Once released, a timer was used to measure the time the animal took to completely right, as defined by the dorsal midline. Three consecutive measurements were recorded for each animal and averaged to produce one data point. Data were analyzed by Student’s *t*-test for pairwise comparisons, and ANOVA tests for all data sets of ≥three comparisons.

### Immunocytochemistry imaging

Immunocytochemistry confocal imaging was performed as described previously (Rushton et al., 2012). L3 larvae were dissected in physiological saline consisting of 128 mM NaCl, 2 mM KCl, 4 mM MgCl_2_, 0.2 mM CaCl_2_, 70 mM sucrose, 5 mM trehalose and 5 mM HEPES (pH 7.1). Preparations were fixed in 4% paraformaldehyde for 10 minutes at RT, and then either processed with detergent (PBTX: PBS+1% BSA+0.2% Triton X-100) for cell-permeabilized studies, or detergent-free (PBS with 1% BSA) for non-permeabilized studies. Primary antibodies used included: Alexa-Fluor-488 goat anti-horseradish peroxidase (HRP, 1:200; Jackson Labs); mouse anti-Fasciclin-II [FASII, 1:10; Developmental Studies Hybridoma Bank (DSHB), University of Iowa]; mouse anti-Discs-large (DLG, 1:250; DSHB); mouse anti-Dally-like-protein (Dlp, 1:4; DSHB) and mouse anti-Wingless (Wg, 1:2; DSHB). Conjugated lectins used included: Peanut agglutinin (PNA, 1:250), *Wisteria floribunda* (WFA-Fitc, 1:250), *Erythrina cristagalli* lectin (ECL, 1:250), *Vicia villosa* agglutinin (VVA, 1:250), Soybean agglutinin (SBA, 1:250) and Wheat germ agglutinin (WGA, 1:250), all from Vector Labs. Secondary antibodies used included: Alexa-Fluor-555 donkey anti-mouse (1:300) and streptavidin Alexa-Fluor-488 conjugate (1:250), both from Invitrogen. Primary antibodies and lectins were incubated at 4°C overnight. Secondary antibodies were incubated at RT for 2 hours. Preparations were mounted in Fluoromount G (Electron Microscopy Sciences).

All mutant and control larvae were dissected, labeled and imaged in parallel. *z*-stacks were taken with a Zeiss LSM 510 META confocal using 40/63× oil-immersion objectives, with sections starting immediately above and ending immediately below the NMJ. Expression analyses of conjugated lectins and anti-HRP were performed using NIH ImageJ software with the threshold function outlining lectin- or HRP-labeled NMJ within FasII-defined synaptic regions. Anti-Dlp and anti-Wg expression analyses were quantified within HRP-defined synaptic regions. Fluorescence was normalized to control values from the same experiment with all imaging parameters kept constant between compared genotypes. For structural analyses, preparations were double-labeled with anti-HRP and anti-DLG, with counts made at muscle 4 in segments A2/3 on the right and left sides. Data were averaged for each animal or hemisegment to produce one data point for expression and structural analyses, respectively. For structural quantification, a bouton was defined as an axon varicosity >1 μm in minimum diameter, and ≥two boutons on one axon defined an NMJ branch. NMJ cumulative length was measured by combining branch lengths, and inter-bouton distances obtained as an average from single branches with ≥six boutons.

### Electrophysiology recording

Two-electrode voltage-clamp (TEVC) electrophysiology was performed as described previously (Rohrbough and Broadie, 2002; Parkinson et al., 2013). Briefly, L3 larvae secured with 3M Vetbond tissue adhesive to sylgard-coated glass coverslips were cut longitudinally along the dorsal midline, internal organs removed and larval bodywall glued down. Peripheral nerves were cut at the ventral nerve cord (VNC). Recordings made at 18°C in physiological saline (see above) from preparations imaged using a Zeiss Axioskop microscope with 40× water-immersion objective. Muscle 6 in segments A2/3 impaled with two 3 M KCl-filled electrodes (~15 MΩ resistance) was clamped (−60 mV) using an Axoclamp-2B amplifier. A glass suction electrode on severed motor nerves stimulated at 0.5 Hz with 0.5 ms suprathreshold stimuli (Grass S88 stimulator). Excitatory junction current (EJC) records filtered at 2 kHz (Clampex) were analyzed with Clampfit software.

### Statistical analyses

Behavioral, functional, structural and fluorescence intensity data were averaged per genotype, calculated as a fold-change relative to the mean control value from the same experiment. Unpaired two-tailed *t*-tests with Welch correction were applied for normally distributed datasets with unequal standard deviations. For data not normally distributed, pairwise comparisons were done with non-parametric Mann-Whitney tests. One-way analysis of variance (ANOVA) was used for parametric multiple comparisons with Tukey-Kramer post-tests. For nonparametric multiple comparisons, Kruskal-Wallis tests were applied with Dunn’s multiple comparisons. All statistical analyses were performed using GraphPad InStat version 3.0 (GraphPad Software). Significance in figures is presented as *P*<0.05 (*), *P*<0.01 (**), *P*<0.001 (***) and *P*<0.0001 (****).
